# Supplementary material for: Contemporary national outcomes of hyperbaric oxygen therapy in necrotizing soft tissue infections
Source: PLoS One. 2024 Mar 21;19(3):e0300738. doi: 10.1371/journal.pone.0300738 (PMC10956790; doi:10.1371/journal.pone.0300738)
Supplement: S1 Table — (DOCX) [file pone.0300738.s001.docx]

|  | ICD9 | ICD10 |
| --- | --- | --- |
| Necrotizing Soft Tissue Infection | 728.86, 040.0, 608.83 | M72.6, N49.3, A48.0 |
| Hyperbaric Oxygen Therapy | 93.95 | 5A05121, 5A05221 |
| Surgical Debridement | 86.04, 86.09, 86.22, 86.28, 83.09, 83.44, 83.45, 83.49 | 0L5xxx, 0L8xxx, 0L9xxx, 0LBxxx, 0LDxxx, 0JCxxx, 0H90xx, 0H91xx, 0H94xx, 0H95xx, 0H96xx, 0H97xx, 0H98xx, 0H99xx, 0H9Axx, 0H9Bxx, 0H9Cxx, 0H9Dxx, 0H9Exx, 0H9Fxx, 0H9Gxx, 0H9Hxx, 0H9Jxx, 0H9Kxx, 0H9Lxx, 0H9Mxx, 0H9Nxx, 0HB0xx, 0HB1xx, 0HB4xx, 0HB5xx, 0HB6xx, 0HB7xx, 0HB8xx, 0HB9xx, 0HBAxx, 0HBBxx, 0HBCxx, 0HBDxx, 0HBExx, 0HBFxx, 0HBGxx, 0HBHxx, 0HBJxx, 0HBKxx, 0HBLxx, 0HBMxx, 0HBNxx, 0H50xx, 0H51xx, 0H54xx, 0H55xx, 0H56xx, 0H57xx, 0H58xx, 0H59xx, 0H5Axx, 0H5Bxx, 0H5Cxx, 0H5Dxx, 0H5Exx, 0H5Fxx, 0H5Gxx, 0H5Hxx, 0H5Jxx, 0H5Kxx, 0H5Lxx, 0H5Mxx, 0H5Nxx, 0H80xx, 0H81xx, 0H84xx, 0H85xx, 0H86xx, 0H87xx, 0H88xx, 0H89xx, 0H8Axx, 0H8Bxx, 0H8Cxx, 0H8Dxx, 0H8Exx, 0H8Fxx, 0H8Gxx, 0H8Hxx, 0H8Jxx, 0H8Kxx, 0H8Lxx, 0H8Mxx, 0H8Nxx, 0J50xx, 0J51xx, 0J54xx, 0J55xx, 0J56xx, 0J57xx, 0J58xx, 0J59xx, 0J5Bxx, 0J5Cxx, 0J5Dxx, 0J5Fxx, 0J5Gxx, 0J5Hxx, 0J5Jxx, 0J5Kxx, 0J5Lxx, 0J5Mxx, 0J5Nxx, 0J5Pxx, 0J5Qxx, 0J5Rxx, 0J80xx, 0J81xx, 0J84xx, 0J85xx, 0J86xx, 0J87xx, 0J88xx, 0J89xx, 0J8Bxx, 0J8Cxx, 0J8Dxx, 0J8Fxx, 0J8Gxx, 0J8Hxx, 0J8Jxx, 0J8Kxx, 0J8Lxx, 0J8Mxx, 0J8Nxx, 0J8Pxx, 0J8Qxx, 0J8Rxx, 0J8Sxx, 0J8Txx, 0J8Vxx, 0J8Wxx, 0J90xx, 0J91xx, 0J94xx, 0J95xx, 0J96xx, 0J97xx, 0J98xx, 0J99xx, 0J9Bxx, 0J9Cxx, 0J9Dxx, 0J9Fxx, 0J9Gxx, 0J9Hxx, 0J9Jxx, 0J9Kxx, 0J9Lxx, 0J9Mxx, 0J9Nxx, 0J9Pxx, 0J9Qxx, 0J9Rxx,  0JB0xx, 0JB1xx, 0JB4xx, 0JB5xx, 0JB6xx, 0JB7xx, 0JB8xx, 0JB9xx, 0JBBxx, 0JBCxx, 0JBDxx, 0JBFxx, 0JBGxx, 0JBHxx, 0JBJxx, 0JBKxx, 0JBLxx, 0JBMxx, 0JBNxx, 0JBPxx, 0JBQxx, 0JBRxx, 0JPSxx, 0JPTxx, 0JPVxx, 0JPWxx, 0JJSxx, 0JJTxx, 0JJVxx, 0JJWxx, 0JN0xx, 0JN1xx, 0JN4xx, 0JN5xx, 0JN6xx, 0JN7xx, 0JN8xx, 0JN9xx, 0JNBxx, 0JNCxx, 0JNDxx, 0JNFxx, 0JNGxx, 0JNHxx, 0JNJxx, 0JNKxx, 0JNLxx, 0JNMxx, 0JNNxx, 0JNPxx, 0JNQxx, 0JNRxx, 0JPSxx, 0JPTxx, 0JPVxx, 0JPWxx, 0JWSxx, 0JWTxx, 0JWVxx, 0JWWxx, 0K50xx, 0K51xx, 0K52xx, 0K53xx, 0K55xx, 0K56xx, 0K57xx, 0K58xx, 0K59xx, 0K5Bxx, 0K5Cxx, 0K5Dxx, 0K5Fxx, 0K5Gxx, 0K5Hxx, 0K5Jxx, 0K5Kxx, 0K5Lxx, 0K5Mxx, 0K5Nxx, 0K5Pxx, 0K5Qxx, 0K5Rxx, 0K5Sxx, 0K5Txx, 0K5Vxx, 0K5Wxx, 0K80xx, 0K81xx, 0K82xx, 0K83xx, 0K85xx, 0K86xx, 0K87xx, 0K88xx, 0K89xx, 0K8Bxx, 0K8Cxx, 0K8Dxx, 0K8Fxx, 0K8Gxx, 0K8Hxx, 0K8Jxx, 0K8Kxx, 0K8Lxx, 0K8Mxx, 0K8Nxx, 0K8Pxx, 0K8Qxx, 0K8Rxx, 0K8Sxx, 0K8Txx, 0K8Vxx, 0KBWxx, 0KB0xx, 0KB1xx, 0KB2xx, 0KB3xx, 0KB5xx, 0KB6xx, 0KB7xx, 0KB8xx, 0KB9xx, 0KBBxx, 0KBCxx, 0KBDxx, 0KBFxx, 0KBGxx, 0KBHxx, 0KBJxx, 0KBKxx, 0KBLxx, 0KBMxx, 0KBNxx, 0KBPxx, 0KBQxx, 0KBRxx, 0KBSxx, 0KBTxx, 0KBVxx, 0KBWxx, 0KT0xx, 0KT1xx, 0KT2xx, 0KT3xx, 0KT5xx, 0KT6xx, 0KT7xx, 0KT8xx, 0KT9xx, 0KTBxx, 0KTCxx, 0KTDxx, 0KTFxx, 0KTGxx, 0KTHxx, 0KTJxx, 0KTKxx, 0KTLxx, 0KTMxx, 0KTNxx, 0KTPxx, 0KTQxx, 0KTRxx, 0KTSxx, 0KTTxx, 0KTVxx, 0KTWxx |

**Supplemental Table 1.** ICD-10 codes included in data retrieval from National Inpatient Sample Database.
