# Supplementary material for: Contemporary national outcomes of hyperbaric oxygen therapy in necrotizing soft tissue infections
Source: PLoS One. 2024 Mar 21;19(3):e0300738. doi: 10.1371/journal.pone.0300738 (PMC10956790; doi:10.1371/journal.pone.0300738)
Supplement: S2 Table — AKI, Acute Kidney Injury. Continuous variables are reported in mean (95% Confidence Interval). (DOCX) [file pone.0300738.s002.docx]

|  | HBOT (n=295) | Control (n=34,963) | P-Value |
| --- | --- | --- | --- |
| Mortality, (%) | ≤10 | 3,267 (9.3) | <0.001 |
| Amputations, (%) | 33 (11.2) | 7,376 (21.1) | <0.001 |
| Acute Myocardial Infarction, (%) | ≤10 | 1,276 (3.6) | 0.18 |
| Pneumonia, (%) | 19 (6.4) | 2,933 (8.4) | 0.11 |
| AKI during admission, (%) | 445 (55.6) | 18,975 (54.3) | 0.88 |
| Discharge Disposition, (%) |  |  | <0.001 |
| Home | 68 (23.1) | 6,008 (17.2) |  |
| Short-term Hospital | ≤10 | 2,128 (6.1) |  |
| Skilled Nursing Facility | 130 (44.1) | 15,533 (44.4) |  |
| Home Health Care | 77(26.1) | 7,529 (21.5) |  |
| Against Medical Advice | ≤10 | 495 (1.4) |  |
| Length of Stay, (Days) | 17.4 (15.4-19.5)* | 16.6 (16.3-16.9) | <0.001 |
| Cost, ($1,000) | 59.2 (53.1-65.3)* | 54.9 (53.7-56.1) | <0.001 |

**Supplemental Table 2.** Results of bivariate analysis comparing outcomes in the hyperbaric oxygen therapy (HBOT) and non-HBOT therapy group in patients with sepsis undergoing surgical intervention for necrotizing soft tissue infection. AKI, Acute Kidney Injury. Continuous variables are reported in mean (95% Confidence Interval).
