# Supplementary material for: Contemporary national outcomes of hyperbaric oxygen therapy in necrotizing soft tissue infections
Source: PLoS One. 2024 Mar 21;19(3):e0300738. doi: 10.1371/journal.pone.0300738 (PMC10956790; doi:10.1371/journal.pone.0300738)
Supplement: S3 Table — (DOCX) [file pone.0300738.s003.docx]

| Covariates included in analysis |
| --- |
| Gender |
| Age |
| Race |
| Hospital Size (by bed number) |
| Hospital Location |
| Insurance (private, government, none) |
| Elixhauser Comorbidity Index |
| Location of Infection (limb or trunk) |
| Clostridial Myonecrosis |
| Cardiac Arrest |
| Myocardial Infarction |
| Acute Respiratory Distress Syndrome |
| Respiratory Failure |
| Prolonged Ventilation |
| Pulmonary Embolism |
| Pneumonia |
| Sepsis |
| Acute Kidney Injury |

Supplemental Table 3. List of covariates included in multivariate analysis
