# Supplementary material for: Contemporary national outcomes of hyperbaric oxygen therapy in necrotizing soft tissue infections
Source: PLoS One. 2024 Mar 21;19(3):e0300738. doi: 10.1371/journal.pone.0300738 (PMC10956790; doi:10.1371/journal.pone.0300738)
Supplement: Attachment — Submitted filename: Response to Reviewers 2.24.24.docx [file pone.0300738.s004.docx]

Dear PLOS One Editorial Board and Reviewers,

Thank you all very much for the opportunity to work together towards publishing our work on “Contemporary National Outcomes of Hyperbaric Oxygen Therapy in Necrotizing Soft Tissue Infections” and especially to the reviewers for their insightful and helpful comments and suggestions on our manuscript. Please find below our responses to each item in red below.

Reviewer #1:

Dear Authors,
I appreciate the opportunity to review your manuscript " Contemporary National Outcomes of Hyperbaric Oxygen Therapy in Necrotizing Tissue Infections."
I have several comments/questions for your consideration:
- Given the complex survey design of the NIS data why is it that the authors have chosen not to apply the survey design features of the NIS to obtain weighted estimates?

- Thank you very much and we really appreciate your experience and insight into the power and utility of the NIS. Weighted data in the NIS survey may offer advantages such as improved representativeness and reduce bias but since we were doing further data manipulation, we, judged the challenges related to model assumptions and potential overfitting outweighed the advantages.

- Please clearly state what variables were included in the adjusted model.

- Thank you for noting this omission, as there was a long list of covariates included, we have included it as a separate supplemental table 3 with the submission and reference this in the methods section (line 118-119)

- Consider the application of a statistical approach to account for potential allocation bias for the hyperbaric oxygen therapy.

- This is a very good point and one we have long considered in our project. As this is a retrospective review of prospectively maintained data, and not a prospective randomized trial, there is a risk of allocation bias owing to individual providers’ experience with HBOT and which patients may or may not tolerate the treatment. Considering this, we constructed our robust multivariate model to account for, and smooth out, any unseen differences to the best of our efforts within the confines of the database. While the NIS is not sufficiently granular to consider individual vitals, we adjusted for demarcation of sepsis and Elixhauser Comorbidity for patient sickness. Additionally, we believe that the extremely large sample size, when combined with multivariate regression, should have a significant smoothing effect on potential biases and confounders.

- Consider including specific comorbid medical conditions ( i.e. Diabetes, CKD, Peripheral artery disease, etc.) in addition to the Elixhauser Comorbidity Index.

-We appreciate your comment. As suggested, we have included the recommended comorbidities in our Table 1 and edited result section (Lines 152-154, Page 8).

- Is hyperbaric oxygen therapy more common to be an inpatient versus an outpatient procedure?

- For this indication, hyperbaric oxygen would be typically given in the inpatient setting. For this investigation we limited our search to only inpatient admissions for that reason. Someone that received HBOT for the indication of necrotizing infection as an outpatient are likely to have been misclassified or receiving treatment for wound care from an instance of necrotizing infection down the line from the acute infection, both situations we aim to exclude from this study by including only inpatient admissions.

Minor comments
- Review Table 3 and Table 4 under discharge disposition. Currently written " Against Medical DEVICE" instead of "Against Medical Advice"

- Thank you very much for catching this, this has been corrected in the revised copy

- line 325: citation method needs to be consistent

- Thank you very much for catching this, this has been corrected in the revised copy

Reviewer #2: Rejected with major improvements in the content.
The topic of the article has been repetitively studied in many articles and there is no new information which is being revealed through this study and it does not seem to have any impactful findings

- We appreciate your insight and review of our work but respectfully disagree. While the topic of hyperbaric oxygen therapy as an adjunctive treatment to antibiotics and prompt surgical debridement has been considered for many years, the clinical equipoise remains in the medical community and there is still no high-grade evidence for any intervention for NSTI. We have added a Cochrane review (Hua 2018) to the background. The vast majority of studies are case series, cohort studies, or retrospective reviews with small sample sizes. Soh et al were the first to use the NIS for this purpose and had a relatively large sample size which showed a benefit associated with HBOT. However, as stated in our introduction, great strides have been made in surgical and critical care of these patients in the ensuing decade, raising the question of whether or not HBOT remains a useful intervention for this at-risk population. Furthermore, while the majority of published studies suggest benefit to HBOT with NSTI, our study shows that it is being only rarely utilized for this indication nationwide. With an upcoming European randomized controlled trial of HBOT for NSTI, our work demonstrates the ‘current state’ in America and offers the largest sample size to date of any single study for the material.

Reviewer #3: The article deals with a very interesting topic, about which there are not many articles covered in this way, which gives the article importance. The topic is presented in an appropriate way and, in my opinion, will contribute to the scientific community in the best possible way.
I am of the opinion that, after checking the statistical data, the paper can be published

- We thank you very much for your consideration for our work and appreciate your time.

Reviewer #4: The authors analysis on utility of Hyperbaric oxygen therapy is a valuable approach. The emphasis should be more on making availability of multiple center of Hyperbaric oxygen therapy and making treatment cheaper rather than concentration on the cost incurred for the treatment. As the author could not collect the data on HBOT duration, number of treatments, treatment pressure, or timing of treatments in relation to time of admission or surgery which are critical data necessary for the management of such patients, the international standards of application of HBOT could be discussed in discussion part for enlightening the reader's knowledge. The Author should get the data of other indications used for HBOT and discuss with the further potentials of HBOT in clinical practice and future publications.

- We very much appreciate the insight and agree with the sentiment that HBOT could be more widely utilized and available for treating a wide variety of conditions, including necrotizing soft tissue infections. As you correctly mention, the limitations of the NIS are such that we are not able to comment on the treatment numbers, depths, or durations used in these patients, unfortunately limiting our ability to speak to the success of certain regimens in practice.
- The Undersea and Hyperbaric Medical Society has published standard of care guidelines for use in common conditions, such as NSTI, however these are largely based on expert opinion and in practice these regimens are often adapted to individual patients or situations (Huang E. UHMS Hyperbaric Medicine Indications Manual, 15th Edition. Best Publishing; 2024). The authors assessed that, for our target audience, discussing the specifics of treatment protocols would be beyond the scope of the paper, especially as we are unable to speak with any certainty on how closely those protocols were followed in our study group(1).
- With regards to other projects, we agree and plan to continue using the NIS to evaluate HBOT indications on a large scale for future works.

Reviewer #5: I like to thank the authors for preparing this manuscript. In this manuscript entitled “Contemporary National Outcomes of Hyperbaric Oxygen Therapy in Necrotizing Soft Tissue Infections”, they present a carefully conducted analysis of a large data pool regarding the in-hospital outcomes of patients admitted with NSTI who received hyperbaric oxygen therapy. The authors have described the results in a very comprehensive manner. However, I have few comments as below:

1. In table 2, to be more explicit, please include % (in addition to <10) of mortality in HBOT column. In cost row and control column, please remove 385 number if it has been added there by mistake.

- Thank you, both have been corrected

2. In table 5, to be mor explicit, please include % (in addition to <10 number) in mortality rows.

- Thank you for this comment. While proportions can convey information about the relative distribution of the variables listed in Table 5, it is crucial to ensure that the proportions do not inadvertently reveal information about specific individuals. This rule for patient privacy has been outlined by the Healthcare Cost and Utilization Project (HCUP) which our used database is maintained by. As suggested, we have included proportion representation (<0.8%) in the Table 5.

1.	Levett D, Bennett MH, Millar I. Adjunctive hyperbaric oxygen for necrotizing fasciitis. The Cochrane database of systematic reviews. 2015;1:CD007937.
